# Aerobic acetone-butanol-isopropanol (ABI) fermentation through a co-culture of *Clostridium beijerinckii* G117 and recombinant *Bacillus subtilis* 1A1

**DOI:** 10.1016/j.mec.2020.e00137

**Published:** 2020-06-11

**Authors:** Yonghao Cui, Jianzhong He, Kun-Lin Yang, Kang Zhou

**Affiliations:** a Department of Chemical and Biomolecular Engineering, National University of Singapore, 4 Engineering Drive 4, Singapore, 117585, Singapore; b Department of Civil and Environmental Engineering, National University of Singapore, 1 Engineering Drive 2, Singapore, 117576, Singapore

**Keywords:** Co-culture, *Bacillus subtilis*, *Clostridium beijerinckii*, Isopropanol, Metabolic engineering

## Abstract

An engineered *B. subtilis* 1A1 strain (BsADH2) expressing a secondary alcohol dehydrogenase (CpSADH) was co-cultured with *C. beijerinckii* G117 under an aerobic condition. During the fermentation on glucose, *B. subtilis* BsADH2 depleted oxygen in culture media completely and created an anaerobic environment for *C. beijerinckii* G117, an obligate anaerobe, to grow. Meanwhile, lactate produced by *B. subtilis* BsADH2 was re-assimilated by *C. beijerinckii* G117. In return, acetone produced by *C. beijerinckii* G117 was reduced into isopropanol by *B. subtilis* BsADH2 via expressing the CpSADH, which helped maintain the redox balance of the engineered *B. subtilis*. In the symbiotic system consisting of two strains, 1.7 ​g/L of acetone, 4.8 ​g/L of butanol, and 0.9 ​g/L of isopropanol (with an isopropanol/acetone ratio of 0.53) was produced from 60 ​g/L of glucose. This symbiotic system also worked when oxygen was supplied to the culture, although less isopropanol was produced (0.9 ​g/L of acetone, 4.9 ​g/L of butanol, and 0.2 ​g/L of isopropanol). The isopropanol titer was increased substantially to 2.5 ​g/L when we increased the inoculum size of *B. subtilis* BsADH2 and optimized other process parameters. With the *Bacillus*-*Clostridium* co-culture, switching from the original acetone-butanol (AB) fermentation to an aerobic acetone-butanol-isopropanol (ABI) fermentation can be easily achieved without genetic engineering of *Clostridium*. This strategy of employing a recombinant *Bacillus* to co-culture with *Clostridium* should be potentially useful to modify traditional acetone-butanol-ethanol fermentation for the production of other value-added chemicals.

## Highlights

• A secondary alcohol dehydrogenase was expressed in *Bacillus subtilis*.
• Acetone-butanol was upgraded into acetone-butanol-isopropanol by *B. subtilis*.
• A mutualistic relationship was established between *B. subtilis* and *C. beijerinckii*.
• Aerobic co-culture of *B. subtilis* and *C. beijerinckii* was achieved.
• *Clostridium* fermentation was improved by introducing a genetically-modified strain.

## 1 Introduction

Butanol is an important chemical that has been widely used as diluent and solvent for producing antibiotics, cosmetics, brake fluid, and hormones (Lee et al., 2008; Li et al., 2013). It has also gained more interest as a biofuel due to its superior performance over ethanol concerning energy content, corrosiveness, volatility, and hygroscopy (Lee et al. 2008, 2012; Wu et al., 2016). Although butanol has been historically produced from petrochemical processes, bio-butanol which can be produced by the genus of *Clostridium* through acetone-butanol-ethanol (ABE) fermentation has become an attractive and sustainable biofuel (Rude and Schirmer, 2009; Lee et al., 2012; Bankar et al., 2013; Li et al., 2013; Youn et al., 2016). However, conventional ABE fermentation is still facing several obstacles even though it has been used for large-scale butanol production in the past (Lee et al., 2012; Bankar et al., 2013). One of the problems is that acetone which accounts for 20–30% of ABE products is regarded as a by-product due to its poor fuel properties and corrosiveness to the rubber parts of the engines (Lee et al., 2012; Youn et al., 2016). Although efforts have been invested to eliminate the production of acetone in ABE fermentation by modifying the acetone synthetic pathway, butanol production usually decreased as well, accompanied by the accumulation of organic acids (Jiang et al., 2009; Sillers et al., 2009; Lehmann et al., 2012). An alternative strategy is to convert acetone into isopropanol which can be utilized as a fuel additive for preparing high-octane gasoline (Rassadin et al., 2006; Survase et al., 2011; Lee et al., 2012). By introducing a secondary alcohol dehydrogenase, acetone produced by *Clostridium* can be reduced into isopropanol without disrupting the acetone synthetic pathway (Dai et al., 2012; Lee et al., 2012). Meanwhile, isopropanol, together with butanol and ethanol produced in this process, can be directly used as biofuel (Lee et al., 2012). Besides, as an alcohol, isopropanol can also be easily converted into esters and recovered from the aqueous phase through liquid-liquid extraction. However, genetic engineering of *Clostridium* is relatively demanding due to the requirement of manipulating the cells under anaerobic conditions (Joseph et al., 2018). The nature of being an obligate anaerobe also requires a strict anaerobic condition during large scale fermentation, resulting in costly anaerobic pretreatment of the medium and equipment (Xin and He, 2013; Wu et al., 2016). A possible solution is biological oxygen depletion inspired by natural microorganism communities in which anaerobes often co-inhabit in aerobic environments together with aerobes (Wu et al., 2016; Mi et al., 2018). Among those genera which can provide respiratory protection for *Clostridium*, *Bacillus* is considered as one of the best candidates due to its fast growth and high tolerance to solvents (Dwidar et al., 2013). A variety of studies have attempted to co-culture *Clostridium* with *Bacillus* so that the ABE fermentation can take place under a condition without anaerobic pretreatment (Tran et al., 2010; Wu et al., 2016; Mi et al., 2018; Oliva-Rodríguez et al., 2019). However, due to the different oxygen requirements of *Bacillus* and *Clostridium*, *Bacillus* was often only used as the oxygen consumer, which limited further applications of these co-culture systems. Therefore, it would be interesting if a co-culture system can switch the anaerobic ABE fermentation into aerobic isopropanol-butanol fermentation and avoid genetic engineering of *Clostridium*.

**Fig. 1:**
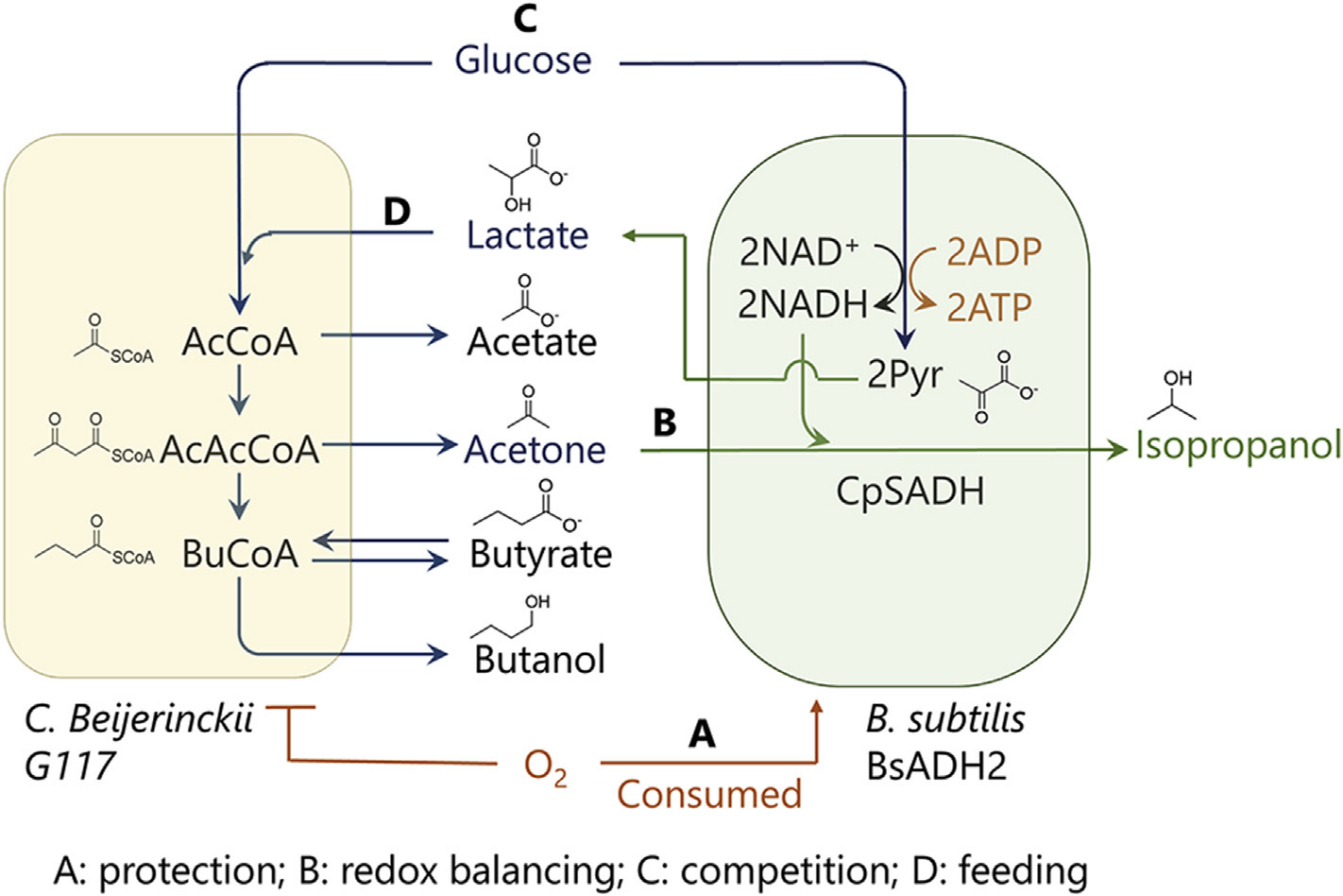
Microbial interactions between the CpSADH-expressing *B. subtilis* (BsADH2) and *C. beijerinckii* G117 in the co-culture. (A) BsADH2 protects *C. beijerinckii* G117 from oxygen. (B) The expression of CpSADH enables BsADH2 to maintain its redox balance by reducing acetone produced by *C. beijerinckii* G117 into isopropanol. (C) BsADH2 and *C. beijerinckii* G117 compete for glucose which is a common carbon source for both strains. (D) lactate produced by BsADH2 was assimilated by *C. beijerinckii* G117. AcCoA: acetyl-CoA. AcAcCoA: acetoacetyl-CoA. BuCoA: butyryl-CoA. Pyr: pyruvate.

In this study, we engineered *B. subtilis* 1A1 to express a secondary alcohol dehydrogenase from *Candida parapsilosis* IFO 1396 (CpSADH) and established a stable symbiotic system composed of this strain and a wild-type *C. beijerinckii* strain G117 that does not produce any ethanol. This co-culture successfully switched the anaerobic acetone-butanol (AB) fermentation into aerobic acetone-butanol-isopropanol (ABI) fermentation without needing any anaerobic pretreatment and reducing agents (Fig. 1).

## 2 Materials and methods

### 2.1 Bacterial strains and plasmids

All strains and plasmids used in this study are listed in Table 1.

**Table 1:** Strains and plasmids used in this study.

Strain or plasmid	Relevant characteristics	Source or reference
Strains		
*C. beijerinckii* G117	Wild type strain	Chua et al. (2013)
Bs1A1	Wild type *B. subtilis* 1A1	BGSC^a^
*E. coli* DH5α		NEB^b^
BsADH1	*B. subtilis* 1A1 Δ*ldh*::operonCm	This study
BsADH2	*B. subtilis* 1A1 harboring pHT01-*cpsadh*	This study
Plasmids		
pHT01	Shuttle vector; Ap^R^, Cm^R^^c^	MoBiTec
pHT01-***cpsadh***	pHT01 carrying *cpadh*, Ap^R^ and Cm^R^	This study
pMB1-***ldh***::operonCm	pMB1 ori Ap^R^ carrying *ldh*::(*cpsadh* Cm^R^)	This study

a Bacillus Genetic Stock Center.

b New England Biolabs.

c Ap^R^, Cm^R^ indicate resistance to ampicillin and chloramphenicol.

### 2.2 Plasmid and strain construction

All primers and barcodes used in constructing the pHT01-*cpsadh* plasmid are listed in Table S2.

The pHT01-*cpsadh* plasmid was constructed according to the GT standard (Ma et al., 2019). The *cpsadh* gene was amplified using P01 and P02 from a plasmid received as a gift. The sequence of *cpsadh* was included in the Supplementary note S1. Then the gene was barcoded with REC3-F and REC4-R. After another round of PCR by using P03 and P04, the fragment was digested by *Bam*HI and *Xba*I. Then the double-digested fragment was ligated with the pHT01 plasmid which was also digested by *Bam*HI and *Xba*I.

The plasmid pMB1-Δ*ldh*:operonCm was also constructed following the GT standard (Supplementary note S2 and Fig. S1).

*Escherichia coli* strain DH5α was used as the recipient in all cloning experiments. After the plasmids were confirmed by using Sanger sequencing, they were used to transform *B. subtilis* 1A1 following the manufacturer’s instruction. The genome-edited *B. subtills* 1A1 was named as BsADH1.The pHT01-*cpsadh* harboring *B. subtills* 1A1 was designated as BsADH2.

### 2.3 Culture media, induction, and growth conditions

#### 2.3.1 *E. coli* DH5α culture medium and growth conditions

*E. coli* DH5α was cultured aerobically at 37 ​°C in Luria-Bertani (LB) medium. Ampicillin (100 ​μg/mL) was added when needed. Agar (15 ​g/L) was added before the sterilization for preparing LB agar plates.

#### 2.3.2 Induction of protein expression in BsADH2

Single colony of BsADH2 was picked from an agar plate with 5 ​μg/mL of chloramphenicol and inoculated into 10 ​mL of 2 ​× ​PY medium (20 ​g/L of peptone, 10 ​g/L of yeast extract, and 10 ​g/L of NaCl, pH 7) with 5 ​μg/mL of chloramphenicol and aerobically incubated at 37 ​°C overnight. Overnight grown cell culture was added into 30 ​mL of fresh 2 ​× ​PY medium with an initial cell density (OD_600_) of 0.15. Then the cells were incubated at 37 ​°C/225 ​rpm. Isopropyl β-d-1-thiogalactopyranoside (IPTG, 1 ​mM) was added when OD_600_ reached 0.7 to 0.8 to induce protein expression. Then the cells were incubated at 37 ​°C/225 ​rpm unless otherwise stated.

#### 2.3.3 *C. beijerinckii* G117 culture media and growth conditions

For the anaerobic culture of *C. beijerinckii* G117, a mineral salts medium (termed as anaerobic medium in this study) was adopted with modification (Xin et al., 2016). The medium contained 0.75 ​g/L of KH_2_PO_4_, 0.75 ​g/L of K_2_HPO_4_, and 5 ​g/L of yeast extract. Besides, 1 ​mL of the trace element solution, 1 ​mL of the Na_2_SeO_3_-Na_2_WO_4_ solution, and 10 ​mg of resazurin were added into 1 ​L of the medium. The trace lement solution contained 2.5 g/L of HCl, 1.5 g/L of FeCl_2_^.^4H_2_O, 0.19 g/L of CoCl_2_^.^6H_2_O, 0.1 g/L of MnCl_2_.4H_2_O, 0.07 g/L of ZnCl_2_, 0.006 g/L of H_3_BO_3_, 0.036 g/L of Na_2_MoO_4_^.^2H_2_O, 0.024 g/L of NiCl_2_^.^6H_2_O, and 0.002 g/L of CuCl_2_^.^2H_2_O. The Na_2_SeO_3_-Na_2_WO_4_ ​solution contained 0.006 g/L of Na_2_SeO_3_^.^5H_2_O, 0.008 g/L of Na_2_WO_4_^.^2H_2_O, and 0.5 g/L of NaOH. Then the medium was flushed with N_2_ and boiled for 20 ​min. After it was cooled down to 25 ​°C, Na_2_S, L-cysteine and dithiothreitol were added as reductants (their final concentrations were 0.2 ​mM, 0.2 ​mM and 0.5 ​mM, respectively). After that, CH_3_COONH_4_ and 2-(N-morpholino)ethanesulfonic (MES) were added to final concentrations of 2 ​g/L and 3.9 ​g/L respectively. After pH was adjusted to 7 using 100 ​g/L of NaOH, the medium was dispensed into serum bottles. Then the bottles were sealed with butyl stoppers and autoclaved at 121 ​°C for 20 ​min.

To prepare the medium without anaerobic pretreatment (named as aerobic medium in this study), all ingredients were mixed in the same concentrations as the anaerobic medium except the reductants and resazurin were omitted. The medium was not flushed with N_2_ during the process. After pH of the medium was adjusted to 7 by 100 ​g/L of NaOH, the medium was filtered through polyethersulfone (PES) membrane whose pore size was 0.22 ​μm. Glucose was supplemented upon inoculation.

*C. beijerinckii* G117 from our laboratory spore stocks was precultured in 50 ​mL of the anaerobic medium supplemented with 60 ​g/L of glucose in a 160-mL serum bottle at 37 ​°C/225 ​rpm for 24 ​h. Then the precultured cells were inoculated into the fresh aerobic medium or anaerobic medium with 60 ​g/L of glucose (inoculum ratio ​= ​10%) and incubated at 37 ​°C/225 ​rpm unless otherwise stated.

#### 2.3.4 Anaerobic acetone reduction by BsADH2 mono-culture

BsADH2 was cultivated at 37 ​°C/225 ​rpm for 8 ​h after induction. Then the cell culture was centrifuged at 20,000 ​g for 10 ​min.

After the supernatant was discarded, the cell pellets were resuspended into 10 ​mL of the anaerobic medium with 60 ​g/L of glucose in a 20-mL serum bottle with a butyl stopper and incubated at 37 ​°C/225 ​rpm. Three grams per liter of acetone was added in the beginning where appropriate. In the control experiment, wild-type *B. subtilis* 1A1 (designated as Bs1A1) was cultivated under the same condition. Samples were taken periodically for analysis.

#### 2.3.5 Co-culture of BsADH2 and *C. beijerinckii* G117

BsADH2 was cultivated at 37 ​°C/225 ​rpm for 8 ​h after the induction. After that, cell pellets collected from 10 ​mL of the cell culture were resuspended into 5 ​mL of the aerobic medium with 60 ​g/L of glucose in a 20-mL serum bottle with a butyl stopper. After being cultured in the anaerobic medium with 60 ​g/L of glucose at 37 ​°C/225 ​rpm for 24 ​h, 500 ​μL of the *C. beijerinckii* G117 ​cell culture was injected into the aerobic medium with BsADH2 above. Then the co-culture was processed at 37 ​°C/225 ​rpm. The pH of the aerobic medium was adjusted to 7 using 100 ​g/L NaOH at 0, 12th and 24th hour. Samples were taken periodically.

In the control experiment, Bs1A1 was firstly cultured following the same procedure with BsADH2 except that no IPTG was added. After that, Bs1A1 was co-cultured with *C. beijerinckii* G117 following the BsADH2-*C. beijerinckii* G117 co-culture protocol described above.

In the aerobic co-culture experiment in the open system, the co-culture was processed in a 50-mL conical tube with a loosened cap at 37 ​°C statically. Other procedures were the same as what was described above.

### 2.4 Determination of number of viable BsADH2 cells in the co-culture

Cell culture from different time points was taken. Then 100 ​μL of cell culture was centrifuged at 20,000 ​g for 2 ​min. After the supernatant was removed, the cell pellets were washed twice with 1 ​mL of sterile DI water. Subsequently, 100 ​μL of sterile deionized (DI) water was used to resuspend the cell pellets. Then the cell suspension was diluted with sterile DI water in series (10^2^ folds, 10^4^ folds, and 10^6^ folds) to enable accurate counting of colonies. Finally, 50 ​μL of each suspension was plated on Lysogeny Broth (LB) agar plate and incubated at 37 ​°C overnight. The number of viable BsADH2 cells was determined by colony counting. Determination of viable Bs1A1 cell number was done following the same procedure as control.

### 2.5 Optimization of process parameters

*C. beijerinckii* G117 was cultured in 5 ​mL of the anaerobic medium with 60 ​g/L of glucose in a 20-mL serum bottle with a butyl stopper at 37 ​°C/225 ​rpm for 24 ​h. After that, cell pellets of the IPTG-induced BsADH2 from 10 ​mL of the cell culture was inoculated into the *C. beijerinckii* G117 culture. After the pH was adjusted to 7 using 100 ​g/L of NaOH, the cells were incubated at 37 ​°C/225 ​rpm. Samples were taken periodically. A control (Bs1A1) was also included.

### 2.6 Protein extraction and analysis

BsADH2 was cultivated at 37 ​°C/225 ​rpm for 8 ​h after induction. Then 10 ​mL of the cell culture was centrifuged at 20,000 ​g for 10 ​min. The supernatant was decanted. The cell pellets were frozen at −20 ​°C for 2 ​h. Subsequently, 1 ​mL of B-PER Complete Reagent (Thermo Scientific) was added to resuspend the cells. After being incubated at room temperature for 15 ​min, the cell lysates were centrifuged at 16,000 ​g for 20 ​min. The supernatant was used for sodium dodecyl sulfate–polyacrylamide gel electrophoresis (SDS-PAGE) based on the manufacturer’s instruction.

In the case of the BsADH1, the cells were cultured in 10 ​mL of the aerobic medium with 60 ​g/L of glucose in a 20-mL serum bottle with a butyl stopper. After being incubated at 37 ​°C for 8 ​h, the cell culture was centrifuged at 20,000 ​g for 10 ​min. Then the cell pellets were frozen at −20 ​°C for 2 ​h and lysed by B-PER. After the cells were lysed, the supernatant was used for SDS-PAGE. A control (Bs1A1) was also included.

### 2.7 Analytical techniques

Cell density was measured by monitoring the optical density (OD) at 600 ​nm with a visible spectrophotometer (Novaspec II, Pharmacia Biotech, Cambridge, England).

Glucose, lactate, acetate, and butyrate were quantified by High Performance Liquid Chromatography (HPLC) (Agilent Technologies, USA) equipped with a refractive index detector (RID) and a variable wavelength detector (VWD, 210 ​nm). A Bio-Rad Aminex HPX-87H column (300 ​mm ​× ​7.8 ​mm) was used. Five micromolar of sulfuric acid was used as the mobile phase at the flow rate of 0.7 ​mL/min.

Butanol, acetone, ethanol, and isopropanol were quantified by gas chromatography (GC, model 7890A; Agilent Technologies, USA) equipped with a Durabond (DB)-WAXetr column (30 ​m ​× ​0.25 ​mm ​× ​0.25 ​μm, J&W, USA) and a flame ionization detector (FID). The oven temperature was initially maintained at 60 ​°C for 2 ​min, increased at 15 ​°C/min to 230 ​°C, and held at 230 ​°C for 1.7 ​min. Helium was used as the carrier gas with a column flow rate of 1.5 ​mL/min (Chua et al., 2013).

## 3 Results

### 3.1 Interaction between CpSADH-expressing *B. subtilis* and *C. beijerinckii* G117

When *B. subtilis* BsADH2 was co-cultured with *C. beijerinckii* G117 in the aerobic medium (as defined in Materials and Methods), BsADH2 grew first, which consumed oxygen in the medium. Meanwhile, lactate was produced by BsADH2 as a product. In this process, glucose was only consumed by BsADH2. After the oxygen was depleted in the peripheral environment, the anaerobic *C. beijerinckii* G117 started to grow under the protection of BsADH2. During the growth of *C. beijerinckii* G117, acetone, butanol, butyrate, and acetate were produced. Meanwhile, the lactate produced by BsADH2 was assimilated by *C. beijerinckii* G117. Besides, acetone was reduced into isopropanol by BsADH2 (Fig. 1). After the oxygen was depleted, glucose was consumed by both BsADH2 and *C. beijerinckii* G117, forming a competitive relationship between the two strains.

### 3.2 Expression of CpSADH in *B. subtilis* 1A1

**Fig. 2:**
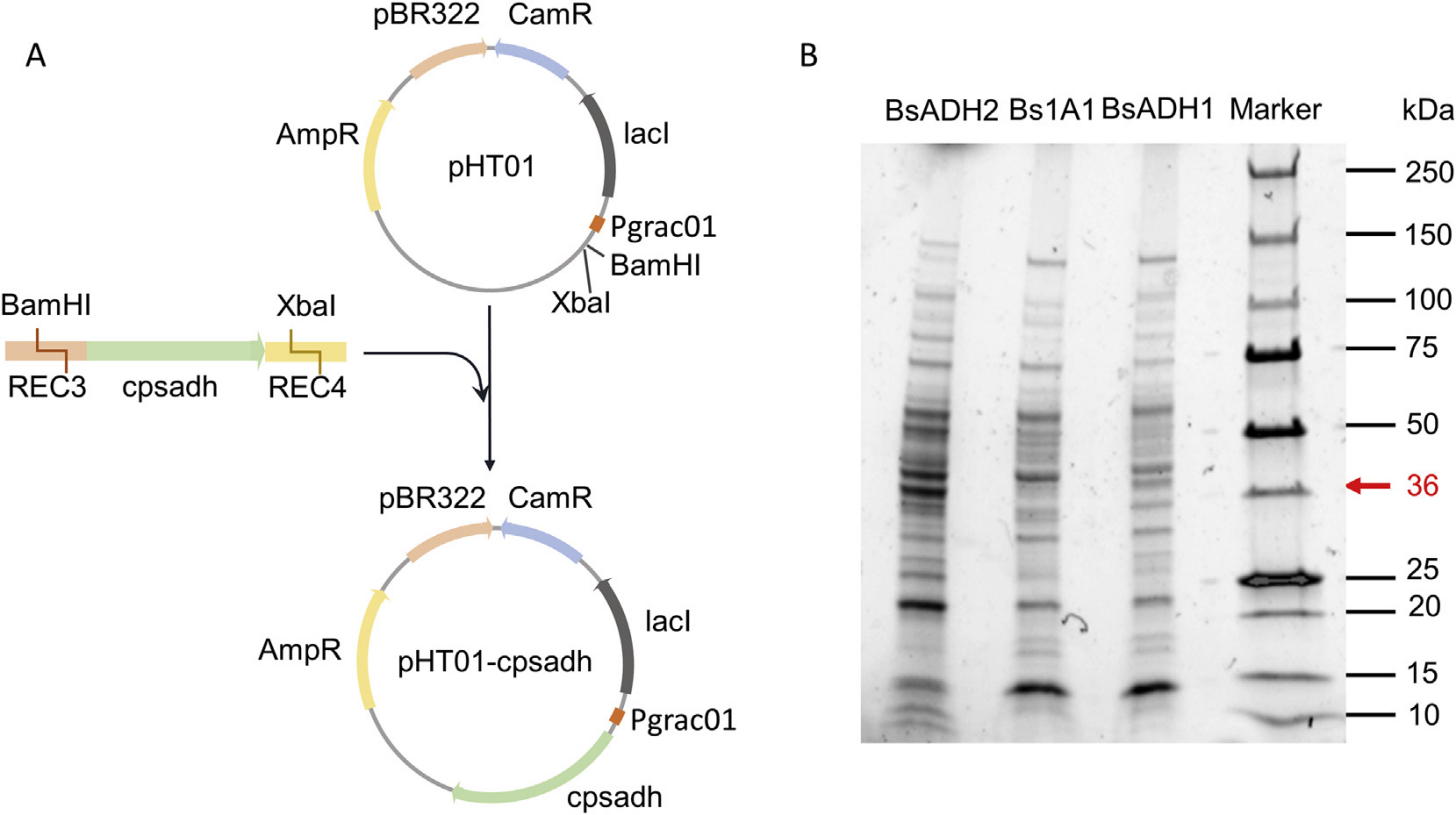
Plasmid construction and expression of CpSADH. (A) Construction of the pHT01-*cpsadh* plasmid. After being barcoded with REC3-F and REC4-R, *cpsadh* was double-digested by *Bam*HI and *Xba*I and ligated with the pHT01 plasmid which was also digested by *Bam*HI and *Xba*I, yielding pHT01-*cpsadh*. (B) SDS-PAGE analysis of the CpSADH expression in BsADH2, Bs1A1 and BsADH1. A thick band at 36 ​kDa (molecular mass of CpSADH) was only seen in the lane of BsADH2.

CpSADH has been widely used in biocatalysis research (Liu and Li, 2019). To investigate the performance of CpSADH of reducing acetone into isopropanol, we constructed a replicative plasmid pHT01-*cpsadh* by cloning *cpsadh* into the pHT01, a commercial *B. subtilis* expression vector (Fig. 2A). Besides, an integrative vector pMB1-*ldh*:operonCm was also constructed as an alternative (Fig. S1). The gene that encodes lactate dehydrogenase (LDH) was chosen as the integration locus because *ldh* promoter was reported to be able to drive the expression of heterologous gene efficiently under non-aerated condition (Romero et al., 2007). We hypothesized that the CpSADH could be expressed by the native *ldh* promoter in the genome-edited strain (named as BsADH1) under the oxygen-limited condition during the co-culture process. These two plasmids were used to transform *B. subtilis* 1A1 separately to compare their performance. After successful insertion of *cpsadh* into *ldh* locus (validated by both colony PCR and Sanger sequencing of PCR products), BsADH1 was cultured in the aerobic medium for 8 ​h in a serum bottle with a butyl stopper to mimic the non-aerated condition that is typically used in *Clostridium* culture (Romero et al., 2007). The strain carrying the replicative plasmid (named as BsADH2) and the parental strain *B. subtilis* 1A1 (termed as Bs1A1, used as a control) were cultured in the same medium with IPTG supplemented when appropriate to induce the protein expression. After the protein expression, cells were harvested and a sodium dodecyl sulfate-polyacrylamide gel electrophoresis (SDS-PAGE) was conducted to assess the expression level of CpSADH. The lane of BsADH2 showed a thick band at 36 ​kDa (the molecular weight of CpSADH), which suggested the successful expression of CpSADH (Fig. 2B). By contrast, no target protein was produced by Bs1A1. Surprisingly, there was no target band when BsADH1 was used, indicating that CpSADH was not expressed even though the sequencing result proved that the integration was successful. This was probably due to the complicated working mechanism of the *ldh* promoter which was beyond the scope of this study. BsADH2 was employed for the rest of the study.

### 3.3 Anaerobic reduction of acetone by mono-culturing BsADH2

After the successful expression of CpSADH, the ability of CpSADH to convert acetone into isopropanol was tested under anaerobic condition. In this experiment, BsADH2 was cultured at 37 ​°C in the anaerobic medium supplemented with 60 ​g/L of glucose and 3 ​g/L of acetone which mimicked the acetone concentration produced by a typical *Clostridium* culture. Besides, BsADH2 was also cultured in the same medium without acetone as a control. Another control experiment with Bs1A1 was also included.

**Fig. 3:**
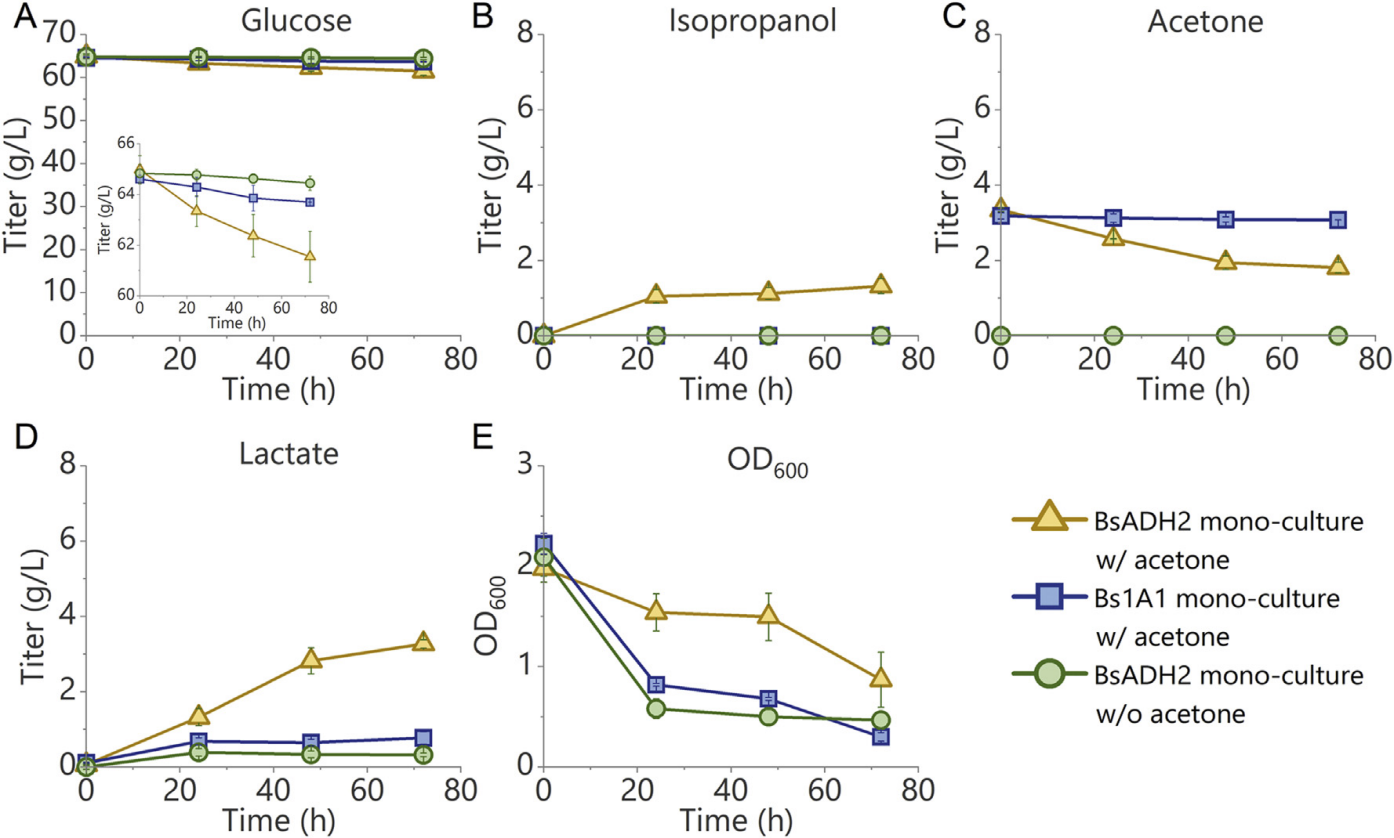
Acetone reduction through the anaerobic mono-culture of BsADH2 and Bs1A1. Time profile of (A) glucose, (B) isopropanol, (C) acetone, (D) lactate, and (E) OD_600_ during the fermentation. Isopropanol was only produced when the *B. subtilis* strain expressed CpSADH and acetone was supplemented. Yellow triangles: BsADH2 anaerobically mono-cultured in the anaerobic medium supplemented with 60 ​g/L glucose and 3 ​g/L acetone. Blue squares: Bs1A1 anaerobically mono-cultured in the anaerobic medium supplemented with 60 ​g/L glucose and 3 ​g/L acetone. Green circles: BsADH2 anaerobically mono-cultured in the anaerobic medium supplemented with 60 ​g/L glucose only. Error bars indicate standard error (n ​= ​3).

In the case of BsADH2 fed with acetone, 1.0 ​g/L of isopropanol was produced after 24 ​h with a productivity of 0.042 ​g/L/h, together with the production of 1.3 ​g/L of lactate from 1.6 ​g/L of consumed glucose (Fig. 3A, B, and D), indicating the successful conversion of some acetone into isopropanol under the anaerobic condition. In this process, BsADH2 converted glucose to lactate at anaerobic condition to generate ATP. Acetone should have served as electron acceptor to allow carbon metabolism to be active beyond the pyruvate branch point. Since 1.6 ​g/L glucose could not provide all the NADH used for both lactate production and acetone reduction under the anaerobic condition, the needed electrons could be derived from further oxidation of glucose beyond pyruvate, which might occur at anaerobic condition in BsADH2 when acetone was used as the electron acceptor. Further cultivating the cells for another 48 ​h led to the production of another 0.3 ​g/L of isopropanol, resulting in total 1.3 ​g/L of isopropanol produced (Fig. 3B). Although the conversion rate was only 39.4% and there was still 1.8 ​g/L of acetone left in the medium (Fig. 3C), this result was comparable with some native isopropanol producers which achieved a conversion rate of 37.2% (Xin et al., 2017). As a comparison, no acetone was reduced when Bs1A1 was cultured anaerobically (Fig. 3C). Interestingly, the lactate titer obtained with Bs1A1 was 0.8 ​g/L, which was lower than what was produced by BsADH2 (3.3 ​g/L). This phenomenon may be due to that glucose uptake was enhanced when acetone was reduced into isopropanol by CpSADH, which was absent in Bs1A1. When BsADH2 was cultured in the same medium without acetone, only 0.3 ​g/L of lactate was produced, suggesting that expressing CpSADH alone was insufficient to trigger the phenotype. Finally, carbon recovery of BsADH2 mono-cultured with acetone, of Bs1A1 mono-cultured with acetone, and of BsADH2 mono-cultured without acetone reached 0.933, 0.736, and 0.865 respectively (Table S1 and Supplementary note S3).

Noteworthily, although the production of lactate kept increasing until the end of the culture, the production of isopropanol in the mono-culture of BsADH2 nearly stopped after 24 ​h (Fig. 3B and D). A possible reason is that the reduction of pyruvate to lactate and reduction of acetone to isopropanol both could accept electrons, the latter of which might not be needed by the cells after the production of lactate started.

*B. subtilis* was reported to be able to grow anaerobically in the presence of glucose/nitrite or glucose/pyruvate (Romero et al., 2007). In our experiment, Bs1A1 could only generate lactate to maintain the redox balance of glycolysis under the anaerobic condition when glucose was fed as the only carbon source. The lactate production cannot sink all the electrons liberated from decarboxylation of pyruvate through pyruvate dehydrogenase, so C2 metabolites may not be obtained anaerobically. We have observed that Bs1A1 became round and smaller under the anaerobic condition (Fig. S2), and the sharp decline of optical density from 2.2 to 0.7 after being cultured anaerobically for 48 ​h (Fig. 3E). As a comparison, the optical density of BsADH2 cultured in the medium with acetone decreased much slower from 2.0 to 1.5 within 48 ​h. By using acetone as the electron acceptor to sink the electrons, pyruvate in BsADH2 may be decarboxylated to produce the C2 metabolites under the anaerobic redox balance constraint, which led to a more active cellular metabolism when BsADH2 was cultured anaerobically. This hypothesis was also supported by the rapid decline of OD_600_ when BsADH2 was anaerobically cultured without acetone as the electron acceptor (Fig. 3E). Being able to reduce acetone anaerobically is vital in the co-culture process because *B. subtilis* needs to maintain an active metabolism for isopropanol production after the oxygen was depleted in the peripheral environment.

In an attempt to improve the production of isopropanol, a few inoculum sizes were tested, but the isopropanol production was not improved (Fig. S3A–B), suggesting that the rate-limiting factor was not the amount of CpSADH.

Culture temperature was reported to be critical in the expression of recombinant protein (Wang et al., 2009). Reducing growth temperature was used as a strategy to improve the expression of the recombinant protein by improving protein folding (Zhou et al., 2012; Mühlmann et al., 2017). Therefore, the cells were cultured at 25 ​°C, 30 ​°C, or 37 ​°C after induction to investigate the effect of temperature on the expression of CpSADH. However, induction at different temperatures did not substantially affect the anaerobic acetone reduction, further suggesting that the rate-limiting factor was not the activity of CpSADH (Fig. S3 C-D).

### 3.4 Switching AB to ABI fermentation by co-culturing *C. beijerinckii* G117 and BsADH2 in the aerobic medium in closed and open systems

After we confirmed that BsADH2 can reduce acetone into isopropanol under the anaerobic condition, we further tested if the strain could reduce acetone produced by *Clostridium* when they were co-cultured*. C. beijerinckii* G117 was chosen as the *Clostridium* strain in the co-culture experiment because it does not produce ethanol, an undesired by-product (Chua et al., 2013). Besides, this strain can also tolerate 5 ​μg/mL of chloramphenicol, which was needed to maintain the replicative plasmid in BsADH2. In addition, *C. beijerinckii* G117 produces negligible amount of acids, which made pH adjustment easier during the fermentation (Chua et al., 2013).

To test if *C. beijerinckii* G117 can grow in the aerobic medium (which did not undergo any anaerobic treatment to remove oxygen) with the help of BsADH2, the two strains were inoculated into the aerobic medium at the beginning of the fermentation. As the induction of protein expression in BsADH2 in the co-culture system might fail due to the anaerobic condition (the promoter was only tested under the aerobic condition), we decided to inoculate high concentrations of induced and aerobically grown BsADH2 cells in the co-culture (initial cell density of BsADH2 ​= ​4 based on OD_600_). To alleviate the effect of lactate production by BsADH2 on pH which might lead to slower growth of *C. beijerinckii* G117, pH was periodically adjusted back to 7 with 100 ​g/L of NaOH during the first 24 ​h (Chua et al., 2013). A control (*C. beijerinckii* G117-Bs1A1 co-culture) going through the same procedure was also included.

**Fig. 4:**
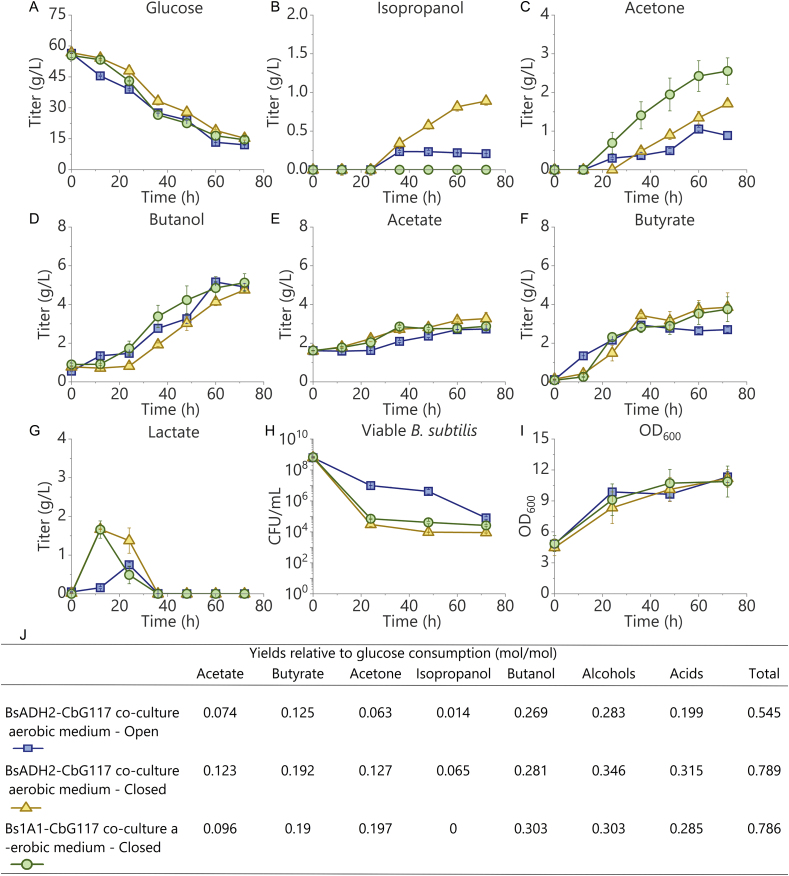
Fermentation results of BsADH2-*C. beijerinckii* G117 and Bs1A1-*C. beijerinckii* G117 co-cultures. BsADH2 produced isopropanol when co-cultured with *C. beijerinckii* G117, even when oxygen continuously diffused to the system. A-I: Time profiles of (A) glucose, (B) isopropanol, (c) acetone, (D) butanol, (E) acetate, (F) butyrate, (G) lactate, (H) number of viable *B. subtilis* cells, and (I) OD_600_ during the co-cultures. J: yields of products. Yields were calculated based on obtained products (mol) per mole of consumed glucose. Alcohols: isopropanol and butanol. Acids: acetate and butyrate. Total: alcohols and acids. Blue squares: BsADH2-*C. beijerinckii* G117 co-culture in the aerobic medium supplemented with 60 ​g/L of glucose in 50-mL conical tubes (open system). Yellow triangles: BsADH2-*C. beijerinckii* G117 co-culture in the aerobic medium supplemented with 60 ​g/L of glucose in serum bottles (closed system). Green circles: Bs1A1-*C. beijerinckii* G117 co-culture in the aerobic medium supplemented with 60 ​g/L of glucose in serum bottles (closed system). Error bars indicate standard error (n ​= ​3).

After the lag phase, *C. beijerinckii* G117 grew rapidly as evidenced by the production of butyrate in the co-culture with BsADH2 (Fig. 4F). After the brief acidogenic phase which produced acetate and butyrate, the cells entered the solventogenic phase from the 24th hour, when butanol and acetone were produced from glucose at 0.081 ​g/L/h and 0.047 ​g/L/h respectively (Fig. 4A, C, D, E and F). During this process, isopropanol was produced along with acetone. The isopropanol concentration stopped to increase after 72 ​h, and reached a final titer of 0.9 ​g/L, together with 4.8 ​g/L of butanol, 1.7 ​g/L of acetone, and 3.9 ​g/L of butyrate (Fig. 4B, C, D, and F). During the last 48 ​h, the productivity of isopropanol was 0.019 ​g/L/h which was 20% of that of butanol (Fig. 4B). Further cultivating the cells for another 48 ​h did not lead to an increase in the butanol and acetone concentrations (data not shown). Finally, the yield of alcohols (butanol and isopropanol) with respect to glucose achieved 0.346 (mol/mol), which was 14% higher than that from the Bs1A1-*C. beijerinckii* G117 co-culture, due to the reduction of acetone (Fig. 4J). The solvent production performance of the co-culture system was comparable with that of the *C. beijerinckii* G117 mono-culture which produced 5.7 ​g/L of butanol and 3.4 ​g/L of acetone under the anaerobic condition (Fig. S4A). As expected, no isopropanol was produced in the anaerobic mono-culture of *C. beijerinckii* G117 (Fig. S4A). Notably, less butyrate was produced in the anaerobic mono-culture of *C. beijerinckii* G117 than what was produced in the co-culture system, which may be due to the adjustment of pH during the co-culture process (Fig. S4B). In the case of Bs1A1 (control) co-cultured with *C. beijerinckii* G117, 5.1 ​g/L of butanol, 2.6 ​g/L of acetone, and 3.8 ​g/L of butyrate were produced (Fig. 4C, D, and F), without the production of isopropanol. This result demonstrated that Bs1A1 was not able to reduce the acetone produced by *C. beijerinckii* G117. When *C. beijerinckii* G117 was cultured in the aerobic medium without *B. subtilis* 1A1 strains (BsADH2 or Bs1A1), it neither produced organic acids/alcohols nor showed cell growth (Fig. S4C). This observation was consistent with the results of other studies (Wu et al., 2016). An interesting phenomenon is that when BsADH2 or Bs1A1 was co-cultured with *C. beijerinckii* G117, lactate was produced in the first 12 ​h. After reaching a plateau at 2 ​g/L at the 12th h, lactate was re-assimilated (Fig. 4G). In comparison to the anaerobic mono-culture of *B. subtilis* 1A1 (BsADH2 and Bs1A1) in which lactate concentration did not decrease, the re-assimilation observed in the co-culture should be attributed to *C. beijerinckii* G117 because some *C. beijerinckii* strains have been reported to utilize lactate to produce butanol and butyrate (Hou et al., 2017; Detman et al., 2019). The re-assimilation of lactate, which was the primary by-product of the *B. subtilis* cells, reduced the waste of carbon source. Besides, the lactate concentration in the Bs1A1-*C. beijerinckii* G117 co-culture decreased more rapidly than that in the BsADH2-*C. beijerinckii* G117 co-culture although the two systems shared similar *C. beijerinckii* G117 growth, suggesting that the BsADH2 may have produced more lactate during the fermentation, which was consistent with what was observed in the anaerobic mono-culture of BsADH2.

To better understand the interactions between the two strains in the co-culture, the number of viable *B. subtilis* (BsADH2 and Bs1A1) cells was determined by counting number of colonies that grew on the aerobically incubated LB agar plates**—***C. beijerinckii* G117 cannot grow aerobically.

Although BsADH2-*C. beijerinckii* G117 co-culture produced isopropanol, it did not contain a larger number of viable *B. subtilis* cells than Bs1A1-*C. beijerinckii* G117 co-culture during the fermentation (Fig. 4H). This was probably due to the absence of acetone during the early stage of the fermentation. After the anaerobic environment was achieved, *C. beijerinckii* G117 experienced a lag phase during which acetone was not produced. However, oxygen was depleted rapidly by BsADH2, which led to the decline of viable BsADH2 cell number due to the lack of electron acceptor (acetone). This was also supported by the rapid decrease of OD_600_ when BsADH2 was anaerobically mono-cultured in the absence of acetone (Fig. 3E). As a result, only a part of BsADH2 population survived to produce isopropanol during the later stage of the co-culture, which might also contribute to the lower productivity of isopropanol in comparison with the anaerobic acetone reduction by BsADH2. By contrast, OD_600_ of the both Bs1A1-*C. beijerinckii* G117 and BsADH2-*C. beijerinckii* G117 co-culture increased over time, which should be due to the growth of *C. beijerinckii* G117 (Fig. 4I).

Conducting the experiments in the medium without any anaerobic pretreatment in serum bottles simplified the experimental procedure. However, the production of gas including H_2_ and CO_2_ by *C. beijerinckii* G117 in a closed system remained a problem. Gas needed to be released regularly to avoid pressure built-up inside the closed system. Therefore, it would be desirable if the co-culture could be maintained in an open system. To achieve this target, we further co-cultured the cells in a 50-mL conical tube whose cap was loosened. This condition was defined in this study as an aerobic condition because oxygen (air) continuously diffused into the system.

The aerobic co-culture of *C. beijerinckii* G117 and BsADH2 in the open system showed similar performance in some aspects as the co-culture in the aerobic medium in the serum bottle. After 72 ​h of cultivation, 4.9 ​g/L of butanol and 2.7 ​g/L of butyrate were produced (Fig. 4D and F). This result suggested that the dissolved oxygen level in the medium under this aerobic condition was zero or very close to zero, which was achieved by the action of BsADH2. Notably, only 0.9 ​g/L of acetone and 0.2 ​g/L isopropanol were obtained, leading to an isopropanol/acetone ratio of 0.22 which was less than half of that obtained from the co-culture in the closed system (Fig. 4B and C). This may be due to the evaporation of acetone and isopropanol which have lower boiling points than butanol. Besides, the respiration of BsADH2 in the open system may also compete with acetone reduction in oxidizing NADH. Additionally, the number of viable BsADH2 cells showed a slower decrease in the open system (Fig. 4H), suggesting that BsADH2 maintained a more active metabolism when oxygen was more accessible. On the other hand, BsADH2 still could protect *C. beijerinckii* G117 from oxygen, leading to the normal growth of *C. beijerinckii* G117 (Fig. 4I).

Compared with other studies only using *Bacillus* as an oxygen consumer, we successfully established a mutualistic relationship consisting of protection and feeding between *C. beijerinckii* and *B. subtilis* (Wu et al., 2016). *B. subtilis* (BsADH2) protected *C. beijerinckii* G117 from the exposure of O_2_ and fed lactate to *C. beijerinckii* G117. In return, *C. beijerinckii* G117 produced acetone for the *B. subtilis* (BsADH2) to better maintain its redox balance. In addition, results from co-culture in the open system proved that the co-culture process we developed here showed the potential as a safer, easier, and more economical way to switch the anaerobic AB fermentation into the aerobic ABI fermentation which does not require the tedious and costly anaerobic pretreatment and troublesome gas-releasing practice.

### 3.5 Optimization of process parameters

As the titer of isopropanol produced in the co-culture system still needed to be improved, we further inoculated BsADH2 into the 24-h anaerobic cell culture of *C. beijerinckii* G117 so that there would be more acetone available when BsADH2 was added.

**Fig. 5:**
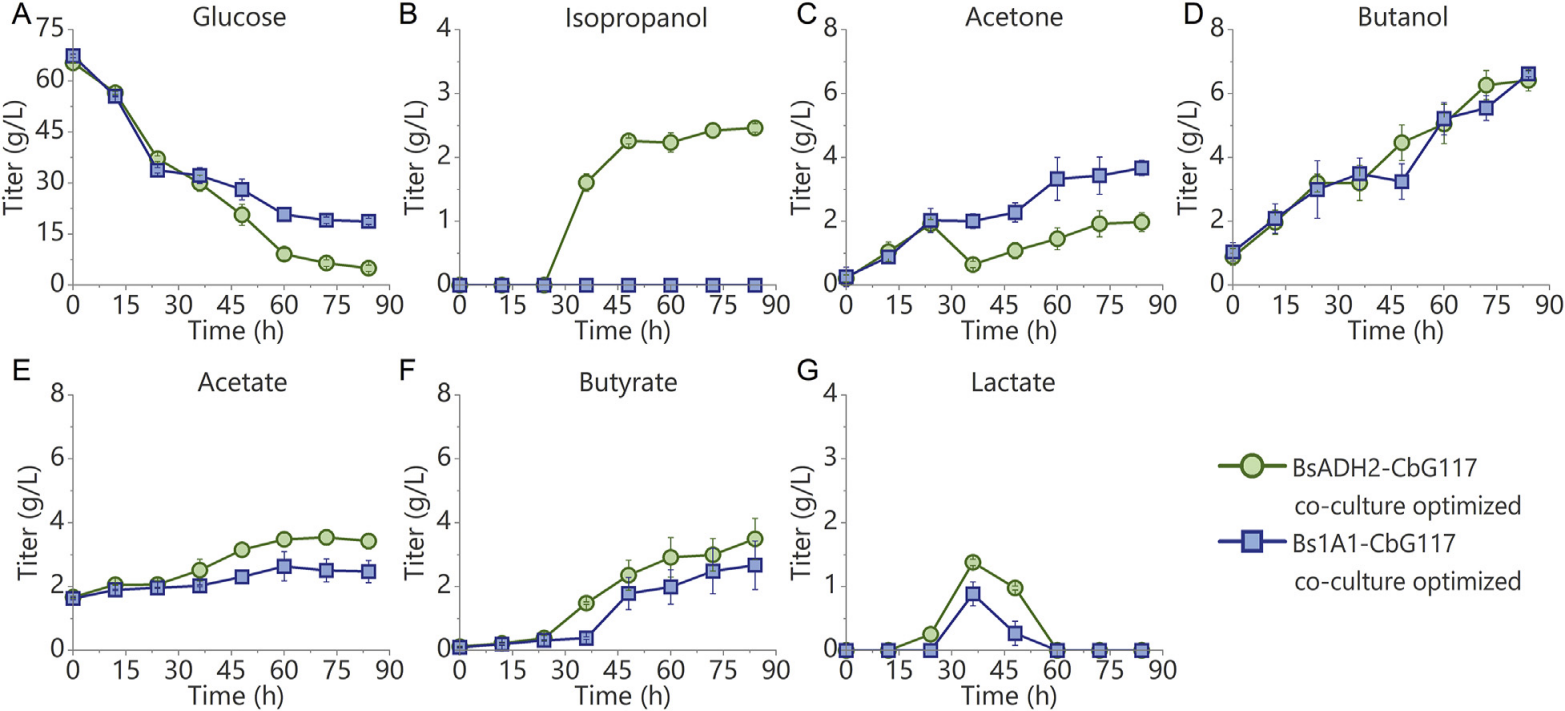
Optimization of process parameters in the co-culture. The optimization was done by adding *B. subtilis* BsADH2 or Bs1A1 into the *C. beijerinckii* G117 culture that had been incubated for 24 ​h. The moment that *C. beijerinckii* G117 mono-culture started was designated as 0 h. Time profiles of (A) glucose, (B) isopropanol, (C) acetone, (D) butanol, (E) acetate, (F) butyrate, and (G) lactate during the fermentation are shown in the figure. Green circles: optimized BsADH2-*C. beijerinckii* G117 co-culture. Blue squares: optimized Bs1A1-*C. beijerinckii* G117 co-culture. Error bars indicate standard error (n ​= ​3).

After the addition of BsADH2, 1.3 ​g/L of acetone was reduced into isopropanol within 12 ​h, and that was more than what was reduced in the aerobic co-culture system (Fig. 5C). Acetate and butyrate were also produced (Fig. 5E and F). Further cultivating the cells for another 12 ​h led to the production of another 0.7 ​g/L of isopropanol from glucose (Fig. 5A, B, C, and D). Afterward, no more isopropanol was produced. This is consistent with the result of the anaerobic acetone reduction experiment by BsADH2 mono-culture that isopropanol was only produced during the first 24 ​h. Overall, 6.4 ​g/L of butanol and 3.5 ​g/L of butyrate were produced after 72 ​h, which was comparable with what were produced by the anaerobic mono-culture of *C. beijerinckii* G117. Meanwhile, 2.0 ​g/L of acetone and 2.5 ​g/L of isopropanol were also obtained (Fig. 5B and C). In comparison, no isopropanol was produced when Bs1A1 was added into the *C. beijerinckii* G117 culture, with similar titers of butanol and butyrate (Fig. 5B, D, and F).

## 4 Discussion and conclusions

The symbiotic system demonstrated in this study is a new approach to improve the *Clostridium* fermentation by introducing an aerobe that can be easily genetically modified. As a proof-of-concept, the co-culture system successfully shifted the anaerobic AB fermentation into the aerobic ABI fermentation, although the acetone produced in the process was not completed reduced into isopropanol. Neither the conversion rate of acetone nor the titer of isopropanol was improved after a few attempts were made to optimize the parameters of the process (Fig. S3). Therefore, improvement of the enzymatic activity is needed and may be achieved by either engineering the CpSADH or evaluating more secondary alcohol dehydrogenases (SADH). Besides, more strains from the genus of *Clostridium* may also be tested in order to improve the titer of products.

In the future, further modifying *B. subtilis* will enable the symbiotic system to have the potential for many other applications. For example, there are many studies trying to produce short-chain esters from ABE fermentation by either expressing an acyl acetyltransferase in *Clostridium* or adding commercial lipases into the medium (Zhang et al., 2017; Noh et al., 2018). Metabolic engineering of *Clostridium* to express acyltransferase led to the production of only 50 ​mg/L of butyl butyrate, which proved the concept and opened the door for further optimization works (Noh et al., 2018). Although the utilization of lipases resulted in the production of 34.7 ​g/L of butyl butyrate by employing an acidogenic *C. tyrobutyricum* strain with the supplementation of butanol, adding commercial lipase and butanol would incur high cost during commercialization (Zhang et al., 2017). Therefore, it would be desired if esters could be produced by co-culturing a *Clostridium* strain with a lipase- and SADH-expressing *B. subtilis*. In this case, *B. subtilis* would provide respiratory protection for *Clostridium* to initiate the ABE fermentation. Meanwhile, *B. subtilis* would maintain its active cellular metabolism to produce lipase by reducing acetone into isopropanol. As a result, isopropanol, butanol, butyrate, and acetate can be further converted into more valuable esters by the lipase. These esters can be easily extracted *in situ* by the utilization of extractant. In addition, further introducing the ability of secreting cellulase, xylanase, and amylase to *B. subtilis* may also enable the expansion of the substrate spectrum of the co-culture system and contribute to the consolidated bioprocessing. Since *B. subtilis* genetic engineering is much easier and faster than the *Clostridium* counterpart, the aforementioned applications with the co-culture would be interesting alternatives to the conventional *Clostridium* mono-culture approach.

Using the co-culture system may face some obstacles. One of them is the usage of the IPTG inducible promoters and antibiotics, which restricts the large-scale application of the process. This could be overcome by utilizing auto-inducible promoters and genome integration vectors. Besides, in order to improve the performance of the co-culture system and enable a better interaction between the two species, several strategies can be explored. For example, controlling oxygen transfer rate (OTR) can be utilized as a strategy to control the microbial interactions in the co-culture system. Due to the different oxygen requirements of the two species, maintaining different oxygen level through the control of OTR can have an impact on the ratio of *B. subtilis* to *Clostridium.* As an important factor, the population ratio of the two specie will further affect the performance of the co-culture system.

As the first co-culture study of an engineered *B. subtilis* strain with *Clostridium*, we successfully switched anaerobic AB fermentation into aerobic ABI fermentation. This study highlighted the potential of using easy-to-engineer *B. subtilis* to functionalize fermentation processes that involve *Clostridium* species. We believe it will inspire more and better co-culture systems involving *Clostridium* species in the future.

## Funding

This work was supported by 10.13039/501100007652Singapore Millennium Foundation (SMF) (R-279-000-516-592) and a Ph.D. scholarship from Singapore Ministry of Education for Yonghao Cui.

## CRediT authorship contribution statement

**Yonghao Cui:** Conceptualization, Methodology, Validation, Writing - original draft, Investigation. **Jianzhong He:** Resources, Writing - review & editing. **Kun-Lin Yang:** Conceptualization, Methodology, Writing - review & editing, Supervision. **Kang Zhou:** Conceptualization, Methodology, Writing - review & editing, Resources, Supervision.

## Declaration of competing interest

The authors declare that they have no known competing financial interests or personal relationships that could have appeared to influence the work reported in this paper.
