# Supplementary material for: Aerobic acetone-butanol-isopropanol (ABI) fermentation through a co-culture of Clostridium beijerinckii G117 and recombinant Bacillus subtilis 1A1
Source: Metab Eng Commun. 2020 Jun 11;11:e00137. doi: 10.1016/j.mec.2020.e00137 (PMC7322341; doi:10.1016/j.mec.2020.e00137)
Supplement: Multimedia component 1 [file mmc1.docx]

**Supplementary information**

**Journal:** Metabolic Engineering Communications

**Title:** Aerobic acetone-butanol-isopropanol (ABI) fermentation through a co-culture of *Clostridium beijerinckii* G117 and recombinant *Bacillus subtilis* 1A1

**Authors:** Yonghao Cui^1^, Jianzhong He^2^, Kun-Lin Yang^1^*, Kang Zhou^1^*

**Affiliations:** ^1^Department of Chemical and Biomolecular Engineering, National University of Singapore, 4 Engineering Drive 4, Singapore 117585, Singapore; email: kang.zhou@nus.edu.sg

^2^Department of Civil and Environmental Engineering, National University of Singapore, 1 Engineering Drive 2, Singapore 117576, Singapore

***Correspondence**: Kang Zhou, Department of Chemical and Biomolecular Engineering, National University of Singapore, Singapore, 117585. Email: [kang.zhou@nus.edu.sg](mailto:kang.zhou@nus.edu.sg) TEL/FAX: +65 6516 3835

Kun-Lin Yang, Department of Chemical and Biomolecular Engineering, 4 Engineering Drive 4, Block E5-02-09, National University of Singapore, 117576, Singapore. Email: [cheyk@nus.edu.sg](mailto:cheyk@nus.edu.sg) TEL/FAX: +65 6516 6614

**Supplementary note S1**

**Sequence of *cpsadh***

5’-ATGTCAATCCCGTCGTCCCAATACGGCTTTGTCTTCAATAAACAGTCTGGTCTGAATCTGCGTAATGATCTGCCGGTCCATAAACCGAAAGCCGGCCAGCTGCTGCTGAAAGTCGATGCAGTGGGTCTGTGTCATTCTGACCTGCACGTGATTTATGAAGGCCTGGATTGCGGTGACAACTACGTTATGGGCCATGAAATTGCAGGTACCGTTGCAGCAGTCGGTGATGACGTCATCAACTATAAAGTGGGTGATCGTGTGGCGTGTGTTGGCCCGAATGGTTGCGGCGGTTGTAAATACTGCCGCGGCGCTATCGATAACGTGTGCAAAAATGCGTTTGGTGATTGGTTCGGCCTGGGTTATGACGGCGGTTATCAGCAATACCTGCTGGTTACCCGTCCGCGCAACCTGAGCCGTATTCCGGATAATGTCTCTGCTGACGTGGCAGCTGCGAGTACCGATGCGGTCCTGACGCCGTATCACGCCATCAAAATGGCACAGGTTTCACCGACCTCGAACATTCTGCTGATCGGTGCCGGCGGTCTGGGCGGTAATGCAATTCAAGTGGCCAAAGCATTTGGTGCGAAAGTTACGGTCCTGGATAAGAAAAAAGAAGCGCGTGATCAGGCGAAAAAACTGGGCGCTGATGCGGTTTATGAAACCCTGCCGGAAAGCATTTCTCCGGGTAGTTTTTCCGCCTGTTTTGATTTCGTGTCAGTTCAGGCAACGTTCGACGTTTGCCAAAAATACGTCGAACCGAAAGGCGTTATCATGCCGGTCGGTCTGGGTGCTCCGAACCTGTCGTTTAATCTGGGTGATCTGGCGCTGCGTGAAATTCGCATCCTGGGCAGCTTCTGGGGTACCACGAACGACCTGGATGACGTGCTGAAACTGGTTTCCGAAGGCAAAGTGAAACCGGTGGTTCGTTCAGCCAAACTGAAAGAACTGCCGGAATATATCGAAAAACTGCGTAACAATGCATACGAAGGTCGCGTCGTCTTTAACCCGTAA -3’

**Supplementary note S2**

**Construction of Integrative Vector pMB1-*ldh*::operonCm**

In order to construct the integrative vector, a GT standard plasmid construction method was adopted (Ma et al., 2019). The chloramphenicol resistance encoding gene was amplified from pHT01 plasmid by using primers PS01 and PS02. The homologous arms *ldh*-HF and *ldh*-HT was amplified from *B. subtilis* genome by using primers PS03 and PS04, PS05 and PS06 respectively. Notably, an RBS barcode was used between *ldh*-HF and *cpsadh*. An I-CeuI barcode was used between *ldh*-HT and AmpR gene so that the plasmid could be linearized before transformed into *B. subtilis* in order to improve the integration efficiency. Barcodes N21, N22, 5UTR3 and N31 were used between CamR and *ldh*-HT, AmpR and pMB1, pMB1 and *ldh*-HF, *cpsadh* and CamR. All the barcodes and primers used in this section can be found in the paper published by Ma et al., 2019 unless otherwise stated.

**Supplementary note S3**

**Carbon recovery calculation of *B. subtilis* anaerobic mono-culture**

Assumptions

1. Cell population did not increase.
2. Isopropanol was all reduced from acetone.

Consumed carbons from glucose (mol/L):

$${\Delta C}_{glucose}=\frac{{\Delta m}_{glucose}}{180}\times6$$

Recovered carbons from lactate (mol/L):

$${\Delta C}_{lactate}=\frac{{\Delta m}_{lactate}}{90}\times3$$

Carbon recovery for glucose:

$${CR}_{glucose}=\frac{{\Delta C}_{lactate}}{{\Delta C}_{glucose}}$$

**Table S1 Carbon recovery calculation**

|  | BsADH2 w/ acetone | Bs1A1 w/ acetone | BsADH2 w/o acetone |
| --- | --- | --- | --- |
| ${\Delta m}_{glucose}$ | 3.44 | 0.91 | 0.37 |
| ${\Delta C}_{glucose}$ | 0.114667 | 0.030333 | 0.012333 |
| ${\Delta m}_{lactate}$ | 3.21 | 0.67 | 0.32 |
| ${\Delta C}_{lactate}$ | 0.107 | 0.022333 | 0.010667 |
| ${CR}_{glucose}$ | 0.933 | 0.736 | 0.865 |

**Table S2 Primers and barcodes used in constructing the pHT01-cpsadh plasmid**

| Primer or barcode | Sequence | Source or reference |
| --- | --- | --- |
| Primer  P01 | 5’-G*TCAATCCCGTCGTCCCAATA-3’ | This study |
| P02 | 5’-A*CGGGTTAAAGACGACGCGAC-3’ | This study |
| P03 | 5’-TCCCAATTAAAGGAGGAAGGA-3’ | This study |
| P04 | 5’-CTGCCCCGGGGACGTC-3’ | This study |
| Barcode |  |  |
| REC3-F | 5’-TCCCAATTAAAGGAGGAAGGATCCAT*G-3’ | Ma et al., 2019 |
| REC4-R | 5’-T*GATCTAGAGTCGACGTCCCCGGGGCAG-3’ | Ma et al., 2019 |

**Figures**


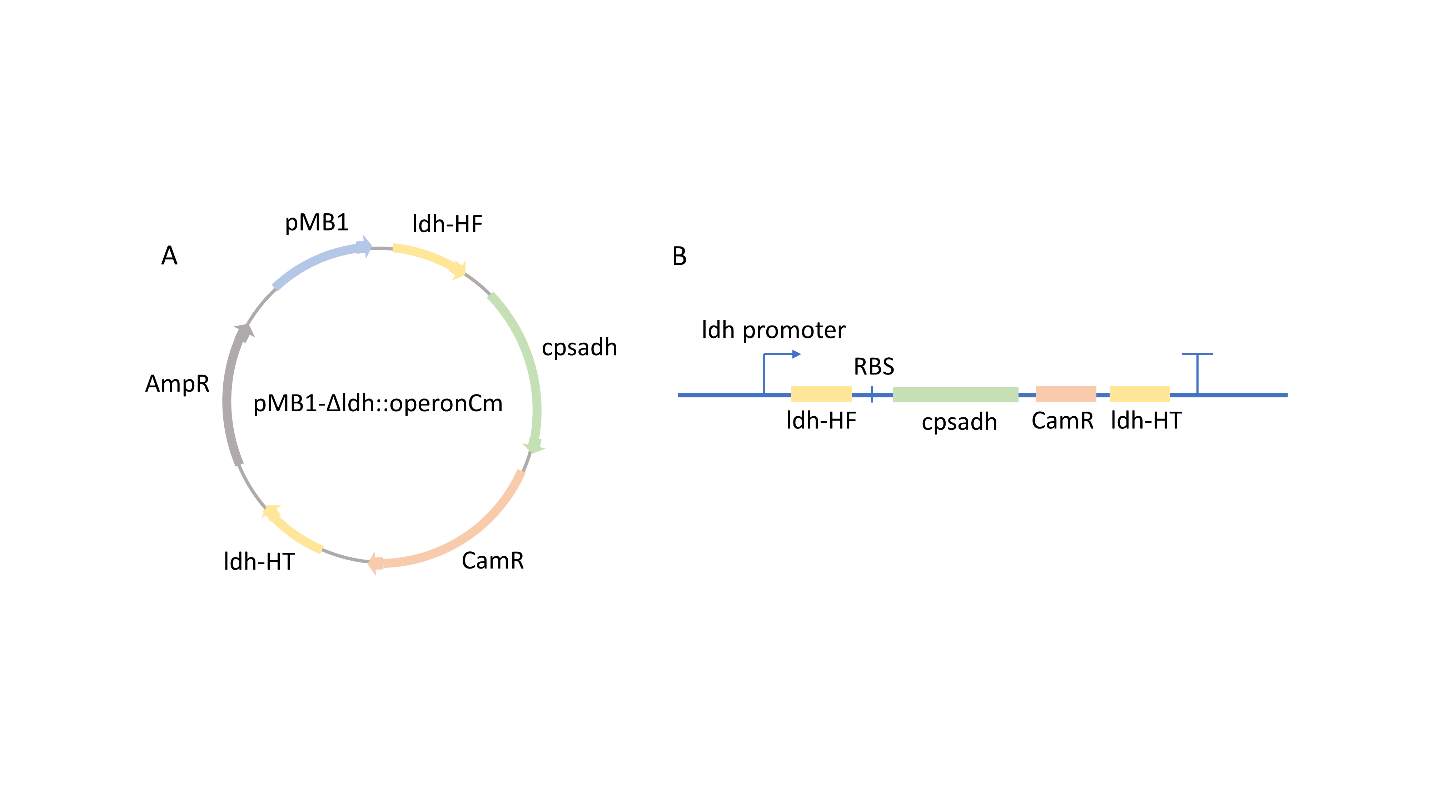


**Fig. S1** (A) Plasmid construction of pMB1-Δ*ldh*::operonCm and (B) illustration of the integration process.


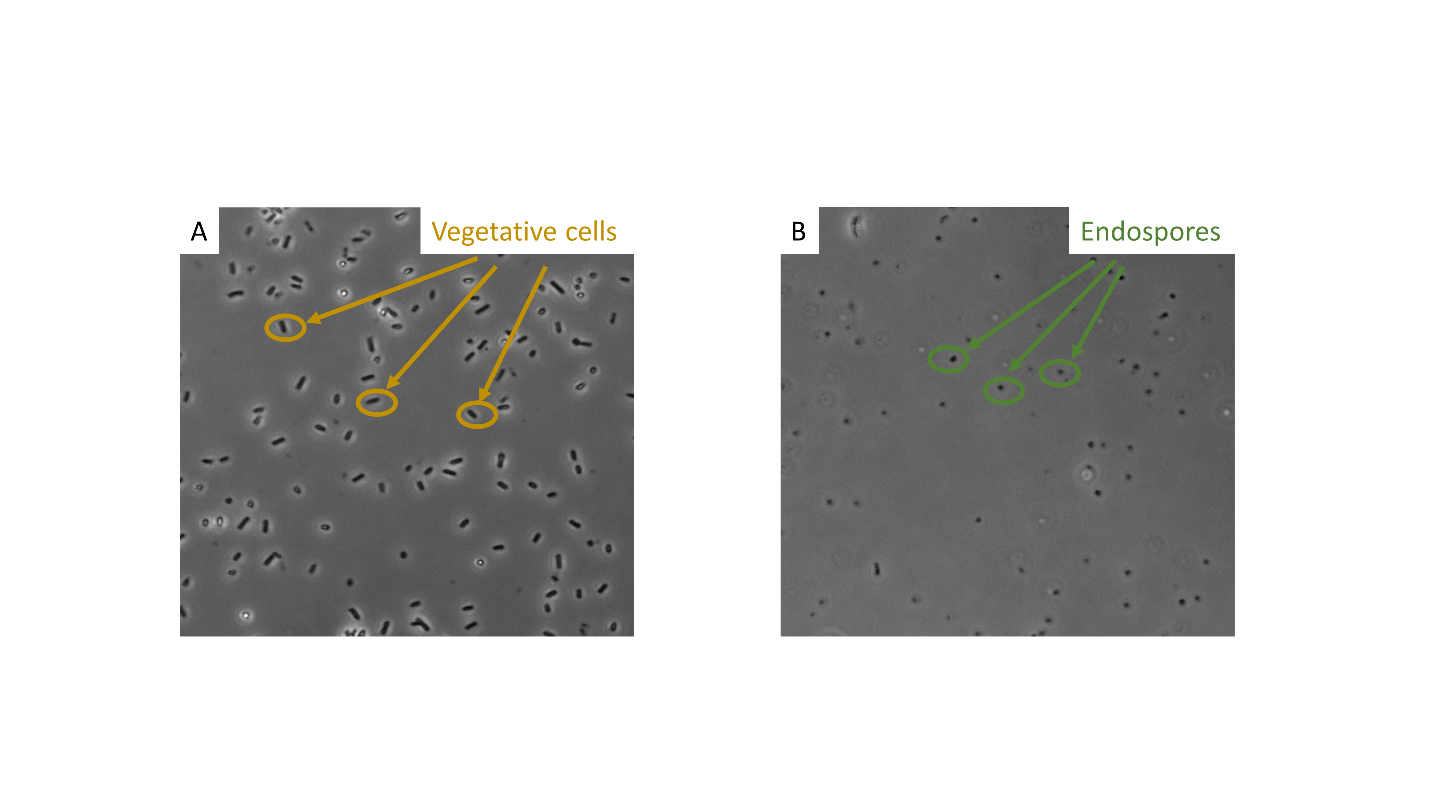


**Fig. S2** Microscopic images of cells from the 48 hours’ anaerobic mono-culture supplemented with 60 g/L of glucose and 3 g/L acetone of (A) BsADH2 and (B) Bs1A1, which were taken by using a phase-contrast microscope with 40 × magnification. The cells in the yellow circles are vegetative cells. The cells in the green circles are endospores.





**Fig. S3** Profiles of anaerobic mono-culture of BsADH2 in the anaerobic medium with 60 g/L of glucose and 3 g/l of acetone. (A) Cells were induced at 37 ^o^C for 8 hours before inoculation. Cell pellets from 15 mL of induced cell culture was inoculated into 5 mL of the anaerobic medium. (B) Cells were induced at 37 ^o^C for 8 hours before inoculation. Cell pellets from 20 mL of induced cell culture was inoculated into 5 mL of the anaerobic medium. (C) Cells were induced at 25 ^o^C overnight before inoculation. Cell pellets from 10 mL of induced cell culture was inoculated into 5 mL of the anaerobic medium. (D) Cells were induced at 30 ^o^C overnight before inoculation. Cell pellets from 10 mL of induced cell culture was inoculated into 5 mL of the anaerobic medium.





**Fig. S4** Batch fermentation profiles of *C. beijerinckii* G117. (A) Glucose and organic solvents concentration of anaerobic mono-culture of *C. beijerinckii* G117. (B) Organic acids concentration of anaerobic mono-culture of *C. beijerinckii* G117. (C) Glucose, organic solvents and acids concentration of *C. beijerinckii* G117 mono-culture in the aerobic medium.
